# Genotypic and Phenotypic Diversity of Staphylococcus aureus Isolates from Cystic Fibrosis Patient Lung Infections and Their Interactions with Pseudomonas aeruginosa

**DOI:** 10.1128/mBio.00735-20

**Published:** 2020-06-23

**Authors:** Eryn E. Bernardy, Robert A. Petit, Vishnu Raghuram, Ashley M. Alexander, Timothy D. Read, Joanna B. Goldberg

**Affiliations:** a Department of Pediatrics, Division of Pulmonology, Allergy/Immunology, Cystic Fibrosis, and Sleep, Emory University, Atlanta, Georgia, USA; b Emory-Children’s Center for Cystic Fibrosis Research, Children’s Healthcare of Atlanta, Atlanta, Georgia, USA; c Department of Medicine, Division of Infectious Diseases, Emory University, Atlanta, Georgia, USA; d Microbiology and Molecular Genetics Program, Graduate Division of Biological and Biomedical Sciences, Laney Graduate School, Emory University, Atlanta, Georgia, USA; e Population Biology, Ecology, and Evolution Program, Graduate Division of Biological and Biomedical Sciences, Laney Graduate School, Emory University, Atlanta, Georgia, USA; University of Washington

**Keywords:** *Pseudomonas aeruginosa*, *Staphylococcus aureus*, cystic fibrosis, interspecies competition, phylogenetic analysis

## Abstract

Staphylococcus aureus has recently overtaken Pseudomonas aeruginosa as the most commonly recognized bacterial pathogen that infects the respiratory tracts of individuals with the genetic disease cystic fibrosis (CF) in the United States. Most studies of S. aureus in CF patient lung infections have focused on a few isolates, often exclusively laboratory-adapted strains, and how they are killed by P. aeruginosa. Less is known about the diversity of S. aureus CF patient lung isolates in terms of both their virulence and their interaction with P. aeruginosa. To begin to address this gap, we recently sequenced 64 clinical S. aureus isolates and a reference isolate, JE2. Here, we analyzed the antibiotic resistance genotypes, sequence types, clonal complexes, *spa* types, *agr* types, and presence/absence of other known virulence factor genes of these isolates. We hypothesized that virulence phenotypes of S. aureus, namely, toxin production and the mucoid phenotype, would be lost in these isolates due to adaptation in the CF patient lung. In contrast to these expectations, we found that most isolates can lyse both rabbit and sheep blood (67.7%) and produce polysaccharide (69.2%), suggesting that these phenotypes were not lost during adaptation to the CF lung. We also identified three distinct phenotypic groups of S. aureus based on their survival in the presence of nonmucoid P. aeruginosa laboratory strain PAO1 and its mucoid derivative. Altogether, our work provides greater insight into the diversity of S. aureus isolates from CF patients, specifically the distribution of important virulence factors and their interaction with P. aeruginosa, all of which have implications in patient health.

## Abstract (precis)

Staphylococcus aureus is now the most frequently detected recognized pathogen in the lungs of individuals who have cystic fibrosis (CF) in the United States, followed closely by Pseudomonas aeruginosa. When these pathogens are found to coinfect the CF lung, patients have a significantly worse prognosis. While P. aeruginosa has been rigorously studied in the context of bacterial pathogenesis in CF, less is known about S. aureus. Here, we present an in-depth study of 64 S. aureus clinical isolates from CF patients, for which we investigated genetic diversity utilizing whole-genome sequencing, virulence phenotypes, and interactions with P. aeruginosa. We found that S. aureus isolated from CF lungs are phylogenetically diverse; most retain known virulence factors and vary in their interactions with P. aeruginosa (i.e., they range from being highly sensitive to P. aeruginosa to completely tolerant to it). Deepening our understanding of how S. aureus responds to its environment and other microbes in the CF lung will enable future development of effective treatments and preventative measures against these formidable infections.

## INTRODUCTION

Cystic fibrosis (CF) is an inherited genetic disease that affects over 70,000 people worldwide and is characterized by mutations in the cystic fibrosis transmembrane conductance regulator (CFTR). When CFTR function is compromised, mucus accumulates in the respiratory tract, creating a breeding ground for chronic bacterial lung infections. These difficult-to-treat infections are the predominant cause of morbidity and mortality for people with CF (1).

Staphylococcus aureus and Pseudomonas aeruginosa are the two most commonly recognized bacterial pathogens associated with chronic lung infections in patients with CF in the United States according to the *Cystic Fibrosis Foundation Patient Registry 2018 Annual Data Report* (2). In fact, S. aureus has recently overtaken P. aeruginosa as the most frequently detected bacterial pathogen in sputum samples from all CF patients in the United States (2). Outside the United States, where continuous antistaphylococcal prophylaxis of CF patients is more common, S. aureus is less frequently isolated (3–7). In addition, according to the United States-based Cystic Fibrosis Foundation’s 2018 annual report, 70% of all individuals with CF were infected with S. aureus and 25% were infected with methicillin-resistant S. aureus (MRSA) (2).

Historically, S. aureus has been isolated from young CF patients, and then P. aeruginosa becomes the dominant species as the patient ages. However, there is a significant number of patients that are coinfected with S. aureus and P. aeruginosa (8). A number of studies (9, 10), including those from our group (11), have shown that coinfection is associated with diminished lung function and more rapid pulmonary decline. The mechanisms responsible for this worsening of disease severity is a topic of intense interest (8, 12, 13); however, exactly what promotes this decreased lung function is not known. Research attempting to understand this health decline has focused mainly on the interactions between these two microbes *in vitro*, and it has been noted that P. aeruginosa readily kills S. aureus (14–17). Furthermore, we have previously found that the mucoid phenotype of P. aeruginosa, which is associated with a chronic infection state, aids in coexistence with S. aureus (18), but this study was limited to one reference isolate of S. aureus from a wound infection (isolate JE2).

To date, the importance of S. aureus in CF remains controversial (13), as P. aeruginosa has historically been recognized as the major pathogen. Perhaps for this reason, the majority of studies on the pathogenesis of S. aureus in CF have focused on its interaction with P. aeruginosa in the context of coinfection. While this is important, understanding the diversity of S. aureus CF isolates themselves as well as their interactions with P. aeruginosa remains understudied. For instance, there have been relatively few large-scale comparative whole-genomic sequence data analyses using S. aureus isolates from patients with CF and none that have observed the corresponding interaction with P. aeruginosa (19–25).

P. aeruginosa and S. aureus are formidable pathogens that are known to alter their virulence phenotypes when shifting from acute to chronic infection of CF patient lungs. Substantial research on P. aeruginosa has shown significant changes in virulence phenotypes after chronic infection in the CF lung; most notable are changes in extracellular products (26). Less is known about S. aureus phenotypic adaptations during chronic infection. Chronic S. aureus infection has been characterized by the development of small-colony variants and a mucoid phenotype (when the bacteria overproduce polysaccharide), both of which aid in persistence (27–33). The prevalence of small-colony variants has been well studied in the United States, as well as other countries (34, 35). However, while substantial work has been done at two CF centers in Germany to better understand the prevalence of mucoid adaptations (34, 36), similar studies in the United States are lacking. Moreover, many S. aureus virulence factors are toxins (37), but few studies investigating toxin production or function in a large number of CF clinical isolates have been performed.

Our results presented here deepen the current understanding of diversity across S. aureus isolates infecting individuals with CF, as well as the isolates’ interactions with P. aeruginosa. We obtained 64 clinical isolates of S. aureus from individuals with CF from both the Cystic Fibrosis Biospecimen Registry (CFBR; a part of Children’s Healthcare of Atlanta and the Emory University Pediatric CF Discovery Core) and Boston Children’s Hospital. Isolates were chosen to obtain a breadth of patient ages, MRSA status, and whether or not these isolates were found with other organisms as detected by the clinical microbiology laboratory. Here, we chose to investigate genotypes and phenotypes believed to be important for S. aureus infection in the CF lung, namely, the staphylococcal protein A (*spa*) and accessory gene regulator (*agr*) types, antibiotic resistance genes, other virulence genes, hemolysis, polysaccharide production, and interaction with P. aeruginosa. Based on what we know of P. aeruginosa adaptation in the CF lung, we hypothesized (i) that S. aureus isolates from CF lung infections were unique from other S. aureus isolates based on genome sequence and (ii) that adaptation to the CF lung environment selected for isolates with less virulence. Although both of these hypotheses were ultimately rejected, we increased our understanding of S. aureus in CF. In terms of hypothesis i, we found that the isolates came from a variety of genotypes common to the United States in general rather than from a CF-specific clade. In terms of hypothesis ii, most S. aureus isolates retained virulence-associated genotypes and phenotypes, although a small number seemed incapable of hemolysis and/or polysaccharide production, suggesting possible adaptation to the lung. Interestingly, we unexpectedly found that not all S. aureus isolates behaved the same with regard to their interactions with P. aeruginosa, signifying the genetic complexity of this phenotype. Together, these studies show the large diversity of S. aureus isolates infecting individuals with CF and the significance that this diversity has for future study and treatment of these infections.

## RESULTS

### Genomic characterization of S. aureus CF clinical isolates, including antibiotic resistance and virulence genes.

To begin to determine the diversity of S. aureus isolates from individuals with CF, we previously reported the genome sequences of 64 S. aureus isolates collected from 50 individuals with CF and the reference isolate JE2 (a total of 65 isolates) as described in the work of Bernardy et al. (38). S. aureus JE2, a derivative of USA300, was used throughout our study as a control non-CF-associated isolate because its sequence and phenotypes were known (39–41). Our CF clinical isolates were obtained from patients with a wide range of ages and were from two different sites (CFBR and Boston Children’s Hospital) (Table 1).

**TABLE 1:** Compilation of metadata, genotypes, and phenotypes from CF clinical isolates of S. aureus and laboratory strain JE2^*a*^

Isolate	Metadata			CC	ST	MRSA/MSSA	Antibiotic resistance or sensitivity to:								*agr* type	*spa* type	Rabbit hemolysis	Sheep hemolysis	Polysaccharide production	Coculture with P. aeruginosa as defined in Fig. 3		
	Patient ID	Patient age (yr)	Coinfection				Aminoglycosides	β-Lactams	Fosfomycin	Glycopeptides	MLS	Multi-drug	Phenicol	Tetracyclines						Coculture group	Fold change with nonmucoid PAO1	Fold change with mucoid PAO1
CFBR_30	CFBR105	53	Yes	5	632	MRSA II	R	R	R	R	R	S	S	S	2	t002	+	+	None	1	9.26E–03	2.41E–01
CFBR_16	CFBR105	53	Yes	5	632	MRSA II	R	R	R	R	R	S	S	S	2	t002	+	+	None	1	1.27E–03	1.78E–01
CFBR_33	CFBR105	54	Yes	5	632	MRSA II	R	R	R	R	R	S	S	S	2	t002	+	+	N	2	8.12E–04	9.30E–03
CFBR_29	CFBR105	53	Yes	5	632	MRSA II	R	R	R	R	R	S	S	S	2	t002	+	+	OP	1	1.34E–03	1.66E–01
CFBR_32	CFBR105	54	Yes	5	632	MRSA II	R	R	R	R	R	S	S	S	2	t002	+	+	None	2	6.54E–05	9.14E–03
CFBR_31	CFBR105	54	Yes	5	632	MRSA II	R	R	R	R	R	S	S	S	2	t002	+	+	None	1	1.50E–03	7.44E–02
BCH-SA-12	11	12	No	5	5	MSSA	R	S	R	S	R	S	S	S	2	t002	+	+	None	1	8.13E–05	2.00E–01
BCH-SA-05	5	36	Yes	5	5	MRSA II	R	R	R	R	R	S	S	S	2	t002	+	+	N	1	1.84E–04	2.03E–01
CFBR_09	CFBR219	20	Yes	5	105	MRSA II	R	R	R	R	R	S	S	S	2	t002	+	+	None	1	1.00E–05	8.00E–01
CFBR_07	CFBR170	56	Yes	5	105	MRSA II	R	R	R	R	R	S	S	S	2	t088	–	–	None	2	1.82E–06	9.14E–03
CFBR_36	CFBR280	12	No	5	5	MRSA II	R	R	S	R	R	S	S	S	2	t1228	+	–	None	1	5.83E–05	1.17E–01
CFBR_34	CFBR280	12	No	5	5	MRSA II	R	R	S	R	R	S	S	S	2	t1228	+	–	N	1	1.17E–03	7.01E–02
CFBR_40	CFBR336	9	Yes	5	5	MRSA II	R	R	R	R	R	S	S	S	2	t067	+	+	None	1	1.05E–03	1.60E–01
CFBR_10	CFBR134	31	Yes	5	5	MRSA II	R	R	R	R	R	S	S	S	2	t777	+	+	N	1	1.28E–03	2.15E–01
BCH-SA-06	5	36	Yes	5	5	MRSA II	R	R	R	R	R	S	S	S	2	t002	+	+	None	1	3.80E–04	6.80E–01
CFBR_22	CFBR150	22	Yes	5	5	MRSA II	R	R	R	R	R	S	S	S	2	t002	–	–	OP	2	6.52E–07	9.16E–03
CFBR_21	CFBR150	22	Yes	5	5	MRSA II	R	R	R	R	R	S	S	S	2	t002	–	–	OP	2	5.63E–07	6.38E–03
CFBR_06	CFBR152	37	Yes	5	5	MRSA II	R	R	R	R	R	S	S	S	2	t002	+	–	OP	1	2.22E–05	1.33E–01
CFBR_24	CFBR201	40	Yes	5	5	MRSA II	R	R	R	R	R	S	S	S	2	t002	+	+	None	3	1.04E–02	1.83E–01
CFBR_12	CFBR148	20	No	5	5	MRSA II	R	R	R	R	R	S	S	S	2	t002	+	–	N	1	7.78E–05	7.78E–02
CFBR_26	CFBR101	23	No	5	5	MRSA II	R	R	R	R	R	S	S	S	2	t002	+	+	OP	2	8.51E–07	8.51E–03
CFBR_25	CFBR101	22	No	5	5	MRSA II	R	R	R	R	R	S	S	S	2	t002	+	+	OP	2	5.71E–07	5.43E–03
CFBR_28	CFBR101	23	No	5	5	MRSA II	R	R	R	R	R	S	S	S	2	t002	+	+	OP	2	3.74E–07	2.65E–03
CFBR_11	CFBR101	24	No	5	5	MRSA II	R	R	R	R	R	S	S	S	2	t002	+	–	None	2	3.20E–06	3.20E–03
CFBR_02	CFBR148	24	Yes	5	5	MRSA II	R	R	R	R	R	S	S	S	2	t002	+	–	None	2	2.92E–06	9.20E–03
CFBR_17	CFBR102	23	Yes	5	225	MSSA	R	S	R	S	R	S	S	S	2	t045	+	+	OP	1	4.77E–04	2.48E–01
CFBR_08	CFBR196	23	Yes	5	225	MRSA II	R	R	R	R	R	S	S	S	2	t045	+	–	N	1	2.58E–05	9.33E–02
CFBR_15	CFBR146	29	No	5	5	MRSA	R	R	R	R	R	S	S	S	2	t002	+	–	N	1	7.50E–05	3.38E–01
BCH-SA-02	2	27	Yes	5	5	MRSA II	R	R	R	R	R	S	S	S	2	t306	–	–	OP	1	2.31E–03	6.60E–01
BCH-SA-01	1	54	Yes	5	5	MRSA II	R	R	R	R	R	S	S	S	2	t002	–	–	OP	1	5.45E–05	1.89E–01
CFBR_20	CFBR149	24	Yes	5	5	MSSA	S	S	R	S	R	S	S	S	2	t548	+	+	OP	1	6.75E–03	3.69E–01
CFBR_01	CFBR122	30	No	5	5	MSSA	R	S	R	S	R	S	S	S	2	t002	+	+	OP	2	2.22E–07	4.56E–04
CFBR_05	CFBR238	28	No	5	5	MSSA	S	R	R	S	R	S	R	R	2	t3673	+	+	N	1	7.50E–04	2.03E–01
CFBR_23	CFBR171	22	Yes	5	5	MSSA	R	R	R	S	R	S	S	S	2	t002	+	+	OP	3	1.17E–02	8.65E–01
CFBR_19	CFBR123	23	Yes	5	5	MSSA	R	S	R	S	R	S	S	S	2	t002	+	+	OP	3	1.31E–02	3.89E–01
JE2	ND	ND	Nd	8	8	MRSA IV	S	R	R	S	S	R	S	S	1	t008	+	+	N	1	1.34E–03	5.73E–01
CFBR_47	CFBR515	16	Yes	8	8	MRSA IV	R	R	R	S	R	R	S	S	1	t008	+	+	N	1	6.19E–03	2.21E–01
CFBR_41	CFBR429	6	No	8	8	MRSA IV	R	R	R	S	R	R	S	S	1	t400	+	–	N	1	7.89E–04	3.52E–01
CFBR_38	CFBR314	6	No	8	8	MRSA IV	R	R	R	S	R	R	S	S	1	t008	+	+	N	1	5.56E–06	1.03E–01
CFBR_43	CFBR447	15	Yes	8	8	MRSA IV	R	R	R	S	R	R	S	S	1	t008	+	+	N	1	9.49E–04	2.76E–01
CFBR_18	CFBR120	42	Yes	8	8	MRSA IV	R	R	R	R	R	R	S	S	1	t008	+	+	N	1	4.09E–03	2.37E–01
CFBR_45	CFBR487	6	No	8	8	MRSA IV	R	R	R	S	R	R	S	S	1	t008	+	+	OP	1	1.21E–03	6.27E–01
CFBR_44	CFBR487	6	No	8	8	MRSA IV	R	R	R	S	R	R	S	S	1	t008	+	+	OP	1	9.43E–04	3.75E–01
CFBR_14	CFBR316	21	No	8	8	MRSA IV	R	R	R	S	R	R	S	S	1	t596	–	–	None	1	4.55E–07	1.59E–01
CFBR_42	CFBR430	6	No	8	8	MRSA IV	S	R	R	S	R	R	S	S	1	t008	+	+	N	1	3.26E–03	4.39E–01
BCH-SA-13	12	34	No	8	8	MSSA	S	R	R	S	S	R	S	S	1	t008	+	+	None	1	6.25E–06	2.09E–01
BCH-SA-15	14	7	No	8	8	MSSA	S	R	R	S	S	R	S	S	1	t5160	+	+	OP	1	3.75E–05	3.44E–01
CFBR_48	CFBR530	17	Yes	8	8	MSSA	R	S	R	S	R	R	S	S	1	t1883	+	+	N	1	7.49E–03	1.04E+00
CFBR_03	CFBR153	26	No	8	8	MSSA	S	R	R	S	S	R	S	S	1	t008	–	–	N	2	2.22E–07	4.17E–03
CFBR_49	CFBR573	2	No	8	1181	MSSA	R	R	R	S	S	R	S	R	1	t334	+	+	OP	1	5.16E–03	9.77E–01
CFBR_27	CFBR101	23	No	8	1181	MRSA II	R	R	R	R	R	R	S	R	1	t334	+	+	OP	2	5.88E–07	2.37E–03
BCH-SA-10	9	22	No	8	8	MSSA	S	R	R	S	S	R	S	S	1	t334	–	–	None	1	2.49E–04	2.34E–01
CFBR_35	CFBR280	12	No	97	97	MSSA	S	S	S	S	S	S	S	S	1	t1236	+	+	N	1	2.44E–03	1.81E–01
BCH-SA-07	6	24	No	97	97	MSSA	S	S	S	S	S	S	S	S	1	t3380	+	+	OP	1	7.50E–04	2.03E–01
CFBR_13	CFBR213	23	No	1	213	MSSA	S	S	S	S	S	S	S	S	1	ND	+	+	N	1	7.92E–05	5.35E–01
BCH-SA-04	4	53	No	1	474	MSSA	S	R	S	S	R	S	S	S	3	t127	+	+	N	1	7.74E–04	4.77E–01
BCH-SA-14	13	17	No	8	72	MSSA	S	R	R	S	S	R	S	S	1	t1346	+	+	None	1	7.03E–04	7.49E–01
BCH-SA-03	3	42	Yes	8	72	MRSA	R	R	R	R	R	R	S	S	1	t9602	+	+	N	2	2.39E–04	3.75E–03
CFBR_39	CFBR322	4	No	30	30	MSSA	R	R	R	S	R	S	S	S	3	t021	–	+	N	1	1.50E–04	1.60E–01
BCH-SA-08	7	8	No	30	30	MSSA	R	R	R	S	R	S	S	S	3	t8114	+	+	None	1	4.57E–05	7.34E–01
BCH-SA-11	10	19	No	30	30	MSSA	R	R	R	S	R	S	S	S	3	t122	–	–	OP	1	7.43E–06	9.23E–02
CFBR_37	CFBR390	11	Yes	30	30	MSSA	S	R	R	S	R	S	S	R	3	t9254	–	+	N	1	4.26E–04	1.53E–01
CFBR_04	CFBR172	24	No	30	37	MSSA	R	R	R	S	R	R	S	S	3	t914	–	–	None	2	2.21E–05	9.12E–03
BCH-SA-09	8	20	No	398	398	MSSA	S	R	S	S	R	S	S	S	1	t1451	+	+	OP	1	1.14E–03	3.57E–01
CFBR_46	CFBR509	17	Yes	45	45	MSSA	S	R	S	S	S	S	S	S	1	t073	+	+	None	3	1.56E–02	3.82E–01

a Coinfection denotes whether a patient was coinfected with P. aeruginosa at the time of S. aureus isolate collection. Roman numeral after “MRSA” denotes the *mec* type. Antibiotic resistance (R) and sensitivity (S) were determined by the ARIBA bioinformatics tool. The “β-Lactams” column describes resistance or sensitivity to β-lactams in addition to methicillin. Phenicol is a class of antibiotics that includes chloramphenicol. MLS stands for macrolides, lincosamides, streptogramins. Glycopeptide resistance was based on *ble*, not *van*, genes. ND, not determined; +, clear hemolysis; –, no hemolysis; N, normal; OP, overproducer. Polysaccharide production is defined in Fig. 2.

**FIG 1:**
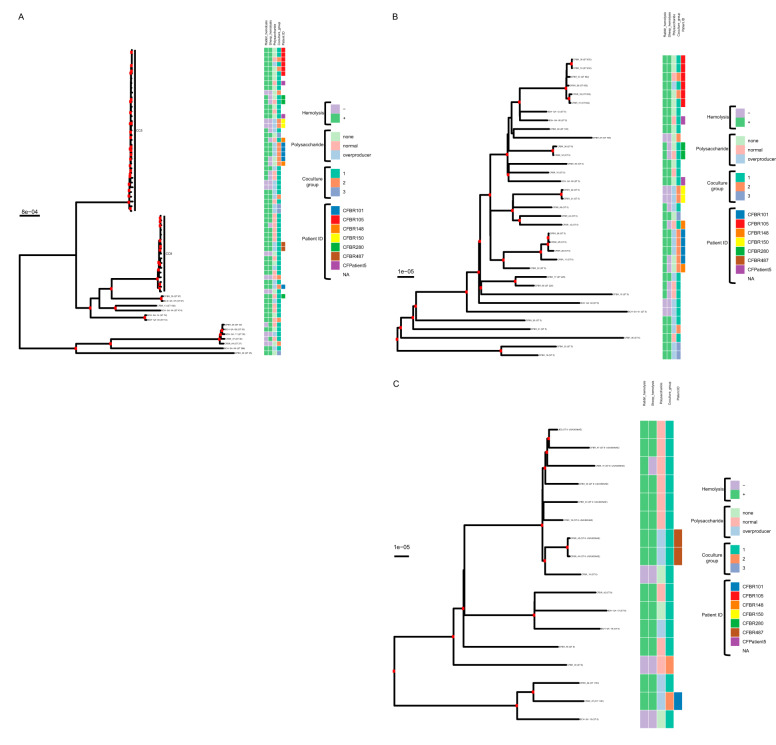
Core genome maximum-likelihood phylogeny of isolates used in this study. Represented is a core genome phylogeny of the 65 isolates in this study produced with IQ-Tree. The tree was built from 1,984 core genes identified by Roary with 25,651 parsimony informative sites and the GTR+F+R2 substitution model. (A) All isolates. Branches with > 90% bootstrap support are indicated with a red dot. The heatmap shows, from left to right, the sheep blood hemolysis phenotype (“+” or “–”); rabbit blood hemolysis (“+” or “–”); whether the isolate was a normal polysaccharide producer, an overproducer, or did not produce polysaccharide; the coculture group (1, 2, or 3); and whether the isolate was one of multiple taken from a patient (CFBR101, CFBR105, CFBR148, CFBR150, CFBR280, CFBR487, CFPatient5, or NA [meaning a single isolate from a patient]). (B and C) Expanded portions of the same tree focusing on CC5 and CC8 isolates, respectively.

We first created a phylogeny to indicate how genetically similar our isolates were to one another. Figure 1 shows that the isolates analyzed here represent 8 phylogenetically diverse clonal complexes (CCs) of the 66 defined previously within the >40,000 S. aureus genomes compiled to date in the publicly available Staphopia database (42). The most common CCs represented in these isolates were CC5 (Fig. 1B) and CC8 (Fig. 1C), which are also the most prevalent hospital-acquired MRSA CCs in the United States (43). In fact, 40 of the 64 clinical isolates (all CC5 or CC8) were MRSA. All CC5 MRSA isolates had the staphylococcal cassette chromosome of *mec* type II (SCC*mec* II), and all CC8 isolates were SCC*mec* type IV (Table 1). Seven isolates were USA300 from the North American lineage, as determined by *in silico* PCR using canonical primers (44, 45). These isolates all contained the Panton-Valentine leukocidin (PVL) toxin genes (*lukSF*), and all but one (CFBR_41) contained type I arginine-catabolic mobile element (ACME) (40); spontaneous loss of ACME cassettes in USA300 strains has occasionally been reported (46). The methicillin-sensitive S. aureus (MSSA) isolates included some CC5 and CC8 isolates and one CC398 isolate (a livestock isolate). Table 1 shows the isolates in the same order as they are represented in the phylogeny (Fig. 1A).

We next analyzed the sequences of all 65 isolates (64 clinical isolates and JE2) for genes associated with resistance to 15 classes of antibiotics, their accessory gene regulatory (*agr*) type, their staphylococcal protein A (*spa*) type, alpha and beta toxin genes, and other known virulence factor genes. The most prevalent predicted antibiotic resistance phenotypes in our set of isolates include resistance to fosfomycin, β-lactams, MLS (macrolides, lincosamides, and streptogramins), and aminoglycosides, which were present in 57, 56, 54, and 49 isolates, respectively (Table 1). No isolates had resistance genes to fusidic acid, rifampin, sulfonamides, thiostrepton, trimethoprim, or tunicamycin. Most isolates (33 out of 65) had 6 resistance genes, while JE2 had 4. The most resistance genes present in one isolate was 8, and only three isolates had no resistance genes. To determine resistance to β-lactams, we looked at genes outside just *mecA*, like *blaZ*; this caused some MSSA isolates to be considered β-lactam resistant (Table 1). Glycopeptide resistance was attributed to bleomycin resistance, not to vancomycin resistance.

We also were interested in resistance to fluoroquinolones because these are common antibiotics used in clinics. Therefore, we screened for fluoroquinolone resistance based on known amino acid changes in GyrA and GrlA (47). Thirty-two of our 65 isolates were predicted to be resistant based on this analysis, with 3 additional isolates possibly having an intermediate resistance phenotype (having only one gene mutated). Thirty-one of these resistant isolates were also MRSA. Three USA300 isolates and JE2 were predicted to be resistant, and these 4 isolates were the only resistant ST8 (sequence type 8) isolates. Twenty out of 25 ST5 isolates and all the ST632 isolates were predicted to be resistant.

The *agr* quorum-sensing system controls multiple virulence factors in S. aureus and is thought to be an essential player in establishing infection (48–50). There are four known types of this system based on mutations and polymorphisms in the histidine kinase and autoinducer peptides (51). Among our isolates, there were 24 *agr* type I, 35 type II, and 6 type III (Table 1). JE2 was known to have *agr* type I, which was confirmed by our sequence analysis. None of our isolates were *agr* type IV, which is the rarest *agr* type.

*Staphylococcus* protein A (*spa*) is implicated in virulence and typing of this gene by identifying the specific repeats in its variable repeat region has historically been used to distinguish between circulating variants of S. aureus during an outbreak (52). Among our isolates, the two most common *spa* types are t002 (25 isolates) and t008 (10 isolates) (Table 1), both of which are common types in the United States (53). JE2 was confirmed as *spa* type t008 in our analysis.

None of our isolates were closely related enough to suggest recent transmission between patients. The maximum average nucleotide identity (ANI) between any two isolates from different patients was 99.9876% (isolates CFBR_45 and CFBR_38), but the minimum within-patient ANI (excluding one outlying isolate, CFBR_11, from the group of isolates from the same patient: CFBR_25, CFBR_26, and CFBR_28) was 99.9936%. CFBR_11 most likely represents an infection with an isolate from a different source compared to others from the same patient.

All 64 clinical isolates had both alpha and beta toxin genes (*hla* and *hlb*, respectively), which has previously been linked to certain classes of infection (37, 54, 55). Compared to a known toxin producer (JE2), 14 isolates had 100% identity to JE2’s alpha toxin, but another 43 had at least 99% identity. For beta toxin, 21 out of 65 isolates had 100% identity with the JE2 beta toxin, and 44 had at least 99% identity.

Finally, we looked at the presence (specifically, percent identity) or absence of 79 known S. aureus virulence factors in the Virulence Factor Database (VFDB) (56, 57) across the genome sequence data from the 64 clinical isolates using the ARIBA tool (58) (see Fig. S1 in the supplemental material). The results were in line with expectations based on the phylogenetic distribution of the isolates. Genes encoding 19 virulence factors were present in all of our isolates, including *adsA*, many of the *cap8* capsule genes (*cap8B*, -*E*, -*F*, -*M*, and -*P*), *ebp*, *esaB*, delta toxin *hld*, *hlgB*, iron sequestration operon *isdA-isdG*, *srtB_1*, and *sspC*. No isolates had *cap8A*, coagulase gene *coa*, adhesin gene *fnbB*, or *sdrD*. Additionally, only three isolates had the enterotoxin *sea* gene. Interestingly, two of those same isolates were also a part of the three isolates that had the toxic shock syndrome toxin 1 (*tsst-1*) gene. The least number of virulence factor genes in a single isolate was 45, and the most was 57 out of the possible 79 tested (Fig. S1).

10.1128/mBio.00735-20.2 FIG S1 Percent identity and absence of 79 known S. aureus virulence factors in 65 isolates in this study. The core VFDB proteome was used to query the database of assembled contigs of the genomes of this study using tblastn. The heatmap shows the percentage identity of the top match for each protein with a match of ≥100 amino acids and ≥40%. If the match fell below these thresholds, it is represented as a white box. The genomes on the *x* axis were ordered by ST and then alphanumerically. Download FIG S1, EPS file, 1.6 MB. Copyright © 2020 Bernardy et al. 2020 Bernardy et al. This content is distributed under the terms of the Creative Commons Attribution 4.0 International license.

### S. aureus CF isolates hemolyze blood and produce polysaccharide.

S. aureus utilizes an arsenal of toxins as virulence factors during infection (37). While detecting toxin genes in S. aureus CF clinical isolates is commonly performed in epidemiology studies (59), the prevalence of toxin production or function in a large set of S. aureus CF clinical isolates has not been performed. Therefore, in order to assess the virulence capabilities of our S. aureus clinical isolates, we measured the presence or absence of clear hemolysis on blood agar plates. Two of the most prominent S. aureus hemolytic toxins are alpha and beta toxin, whose production can be tested by observing clear hemolysis on rabbit and sheep blood agar plates, respectively (60–62). Of the 65 isolates tested, 44 hemolyze both rabbit and sheep blood (Rabbit +/Sheep +) (Table 2), confirming alpha and beta toxin production, while 10 could not hemolyze either blood agar (Rabbit −/Sheep −) (Table 2). The remaining 11 isolates were positive for only one type of blood hemolysis (Rabbit +/Sheep − or Rabbit −/Sheep +) (Table 2). The presence/absence of hemolysis is also represented on the heatmap in Fig. 1. Even though all isolates had both alpha and beta toxin genes present, the activity was not apparent in some of these isolates, likely due to mutations elsewhere in the genome (63).

**TABLE 2:** S. aureus CF isolates positive or negative for clear hemolysis on blood agar plates

Group^*a*^	No. of isolates showing hemolysis on rabbit or sheep blood agar			
	Rabbit +/Sheep +	Rabbit −/Sheep −	Rabbit +/Sheep −	Rabbit −/Sheep +
1	32	5	7	2
2	8	5	2	0
3	4	0	0	0

Total	44	10	9	2

a Groups are defined in the text on the basis of the isolates’ interaction with P. aeruginosa.

**FIG 2:**
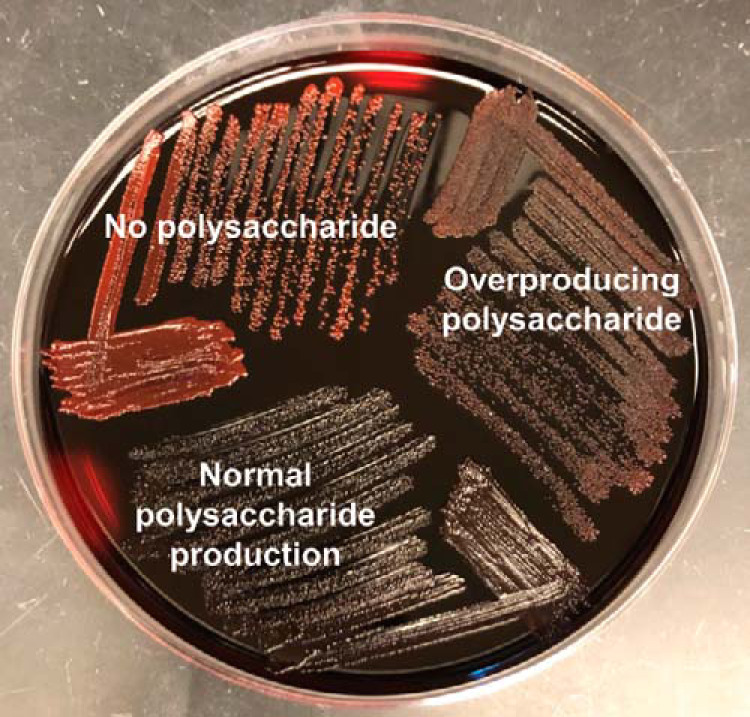
Plating on Congo red agar shows three phenotypes. One representative isolate of each phenotype is shown. Isolates classified as having no polysaccharide are bright red in color and smooth or shiny in appearance. Isolates classified as having normal polysaccharide production are much darker in color than those with no polysaccharide production (many were black, as shown in this figure); these isolates are also smooth or shiny in appearance. Finally, isolates classified as overproducing polysaccharide are also darker than the no-polysaccharide isolates but have a rough or matte appearance.

Another important aspect of S. aureus physiology associated with virulence and persistence is the mucoid phenotype characterized by an overproduction of the polysaccharide poly-*N*-acetyl-β-(1,6)-glucosamine (PNAG) (64–66). Therefore, the polysaccharide production of each isolate was assessed by growing it on a Congo red agar (CRA) plate as described previously (64, 67). Results were interpreted by observing both the color and the appearance of colonies (smooth versus rough) on plates. Genetically defined isogenic mutants of S. aureus strain MN8 served as controls (66). We observed three different phenotypes, and an example of each of these is plated on CRA in Fig. 2. Among the 65 isolates tested, there was an even split among nonproducers (20 out of 65), normal polysaccharide producers (23 out of 65), and overproducers (22 out of 65) (Table 1; Fig. 1). Overall, 45 isolates (69.2%) were capable of producing polysaccharides to some degree, suggesting that this phenotype is conserved in S. aureus CF clinical isolates.

The *ica* operon is known to be responsible for this phenotype; specifically, a 5-bp deletion upstream of the *icaA* gene is known to confer a mucoid or overproducer phenotype (64, 66). Interestingly, none of these isolates had this deletion, indicating the presence of other mutations that cause this phenotype. This observation is consistent with a recent study by Lennartz et al., where the authors identified a small number of S. aureus CF mucoid isolates collected at CF centers in Germany that also did not have this mutation (36).

We were also interested in another phenotype implicated in S. aureus persistence, the presence of small-colony variants (27–30). However, none of our isolates were identified as small-colony variants when they were isolated by the clinical microbiology laboratory or in our laboratory.

### S. aureus CF isolates fall into three distinct groups based on interactions with P. aeruginosa.

Previously, it has been shown that nonmucoid PAO1 kills S. aureus lab isolate JE2, while mucoid PAO1 does not (18). Therefore, we were curious whether this trend would be maintained with CF clinical isolates of S. aureus, and consequently, all of our 65 S. aureus isolates were assessed in a coculture assay with both nonmucoid and mucoid PAO1. While a number of different techniques have been used to look at how P. aeruginosa and S. aureus survive in coculture (18, 68–72), we have developed a simple, repeatable, well-controlled, quantitative assay for monitoring these interactions *in vitro*. This coculture assay allows for these bacteria to come in contact with one another on a solid surface as they might in a biofilm in the CF lung environment. We are also able to grow each species by itself under the same conditions in order to better understand how the bacterium’s survival changes when it is grown in coculture versus alone. While we believe that this protocol is a novel method to investigate this interaction in a consistent and reproducible way, we recognize that it may not exactly replicate conditions *in vivo*.

**FIG 3:**
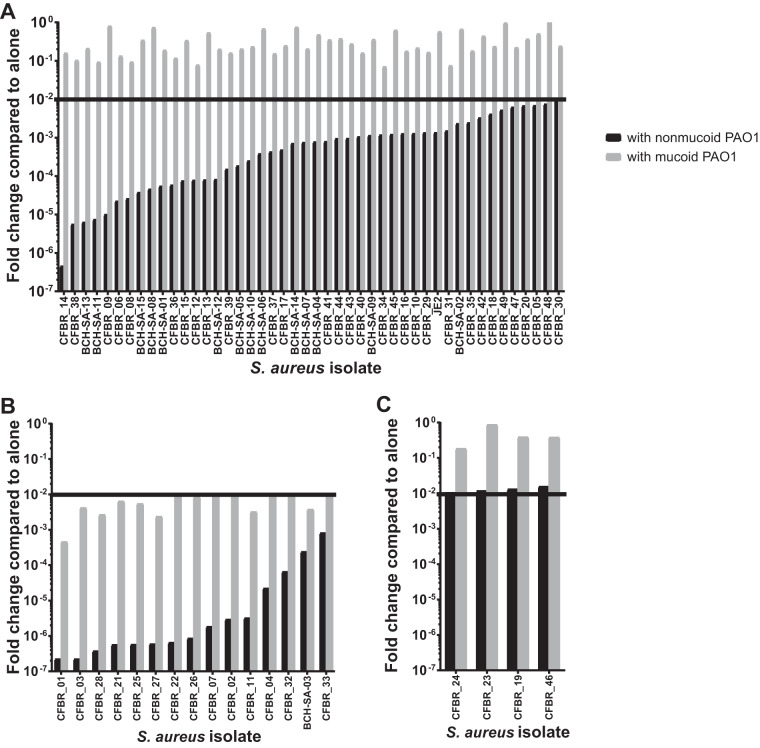
Coculturing S. aureus CF isolates with nonmucoid and mucoid PAO1 revealed 3 interaction groups. Shown are fold changes of each S. aureus isolate after coculture with both nonmucoid (black bars) and mucoid (gray bars) PAO1. Fold change was calculated by dividing the number (per milliliter) of CFU of each S. aureus isolate grown with nonmucoid PAO1 or with mucoid PAO1 by the number of CFU of each S. aureus isolate grown alone. A fold change of <10^−2^ was considered to represent significant killing, as shown by the horizontal black line, denoted a “killing line” in the text. (A) Group 1 isolates (nonmucoid kills) have black bars below 10^−2^, while all gray bars are above this threshold. There were 46 isolates that fit into this interaction group, including the previously tested lab isolate JE2. (B) Group 2 isolates (both kill) have both black and gray bars below the 10^−2^ threshold. Fifteen isolates are in this interaction group. (C) Group 3 isolates (neither kills) have both black and gray bars above the 10^−2^ threshold. Four isolates are in this group. The averages from technical triplicates of one experiment representative of the three biological replicates performed are shown.

To determine whether or not each S. aureus isolate is killed in the presence of P. aeruginosa, the fold change of S. aureus grown in coculture with P. aeruginosa compared to when it grew alone under the same conditions was calculated (Fig. 3). A fold change of less than 10^−2^ (or a >100-fold decrease in the number of CFU per milliliter when the isolate was grown with P. aeruginosa) was considered significantly killed (Fig. 3, horizontal black line), based on other coculture assays that measure killing (73). This definition allowed us to assign each isolate into a group based on how it interacted with nonmucoid PAO1 and mucoid PAO1. Group 1 isolates are those that fit with the previous trend observed and are killed only by nonmucoid PAO1 (Fig. 3A). We observed a range in fold changes, but each fit this trend; the black bars designating fold changes from levels of growth with nonmucoid PAO1 are all below the killing line, while the gray bars designating fold changes from levels of growth with mucoid PAO1 are all above the killing line. Most isolates (46 out of 65), including previously tested isolate JE2, fit in this group (Fig. 3A). Group 2 isolates were those killed by both nonmucoid and mucoid PAO1 isolates (15 out of 65) (Fig. 3B), for which both black and gray bars are below the killing line. Finally, group 3 isolates were those not killed by either PAO1 strain (4 out of 65) (Fig. 3C), for which both bars are above the killing line. Coculture group is also represented on the heatmap in Fig. 1 (“Coculture_group” column). For the remainder of the paper, any mention of coculture group will also have “nonmucoid kills” for group 1, “both kill” for group 2, and “neither kill” for group 3 to help remind the reader of these phenotypic definitions.

None of the S. aureus isolates that we tested affected the growth of either the nonmucoid or the mucoid PAO1 strain in this assay (Fig. S2). We also determined that the decrease in survival when S. aureus is grown with P. aeruginosa is due to killing, not growth inhibition. This was shown by performing the same coculture assay, but instead of taking one time point at 24 h, we measured numbers of CFU of S. aureus per milliliter alone and with both nonmucoid and mucoid P. aeruginosa at multiple time points. We observed that S. aureus grows well with P. aeruginosa until approximately 12 h, when the cell numbers of S. aureus grown with nonmucoid PAO1 drop dramatically (Fig. S3), suggesting that the fold changes we see are, in fact, killing and not due to growth inhibition. Finally, these S. aureus isolates did not inherently have different growth patterns. As shown in Fig. S4, all S. aureus isolates when grown alone grow to approximately the same number of CFU per milliliter (∼10^9^) over 24 h under the coculture assay conditions. Therefore, we conclude that the differences in survival of the various S. aureus isolates when grown with P. aeruginosa are not due to inherent differences in growth.

10.1128/mBio.00735-20.3 FIG S2 No effect on P. aeruginosa when cocultured with S. aureus for 24 h. Average numbers of CFU per milliliter of nonmucoid (top graph, black bars) and mucoid (bottom graph, grey bars) PAO1 after 24 h of coculture with each S. aureus isolate. No S. aureus isolate tested affected P. aeruginosa growth. Download FIG S2, EPS file, 0.6 MB. Copyright © 2020 Bernardy et al. 2020 Bernardy et al. This content is distributed under the terms of the Creative Commons Attribution 4.0 International license.

10.1128/mBio.00735-20.4 FIG S3 S. aureus group 1 isolate grown over time with and without nonmucoid or mucoid PAO1. The average number of CFU per milliliter of a group 1 (nonmucoid kills) S. aureus isolate, JE2, grown alone (orange), with nonmucoid PAO1 (black), and with mucoid PAO1 (gray) over 24 h using the same coculture assay procedure as used for Fig. 2. All three experimental groups grew well initially, and then the black line (S. aureus grown with nonmucoid PAO1) substantially decreased at around 12 to 14 h, while the other S. aureus populations survived, suggesting that changes seen in S. aureus during this coculture assay are due to killing and not growth inhibition. Download FIG S3, EPS file, 0.6 MB. Copyright © 2020 Bernardy et al. 2020 Bernardy et al. This content is distributed under the terms of the Creative Commons Attribution 4.0 International license.

10.1128/mBio.00735-20.5 FIG S4 All S. aureus isolates grow well alone. The average number of CFU per milliliter of each S. aureus isolate “alone” controls during coculture assay (these exact numbers were used to calculate the fold changes in Fig. 2). You can see little variation among the numbers of CFU per milliliter of the isolates after 24 h under coculture assay conditions, suggesting that the fold change seen in Fig. 2 is not an artifact of differences in S. aureus growth levels. Download FIG S4, EPS file, 0.7 MB. Copyright © 2020 Bernardy et al. 2020 Bernardy et al. This content is distributed under the terms of the Creative Commons Attribution 4.0 International license.

## DISCUSSION

### Phylogenetic relatedness to genotypes and phenotypes.

To understand the diversity present in this set of 64 S. aureus CF clinical isolates and the reference isolate JE2 (65 total), we sought to combine our genotypic and phenotypic data, as well as metadata obtained from the clinical microbiology lab. Most of our isolates belonged to CC5 and CC8, which is consistent with the most commonly acquired hospital MRSA isolates, possibly suggesting nosocomial acquisition. In Table 1, the isolates are in the order placed in the phylogeny in Fig. 1, and the results of the tested phenotypes, as well as whether the isolates are longitudinal, are represented in a heatmap in Fig. 1. Therefore, we can observe whether genotypes and phenotypes are linked based on full-genome sequence relatedness. As expected, isolates with the same clonal complexes and sequence types, as well as the MRSA/MSSA phenotype, *agr* type, and *spa* type, cluster together (Table 1). When we observed the presence/absence of virulence factors, all 8 isolates that had Panton-Valentine leukocidin (PVL) genes, *lukS* and *lukF*, including JE2 (see Fig. S1 in the supplemental material), were clustered together in the CC8 group (Fig. 1C). A similar clustering was seen when we observed the 3 isolates that had the toxin genes *sea* and *tsst-1*.

When comparing phylogenetic relatedness and observed phenotypes, we revealed some interesting connections. While isolates with rabbit and sheep hemolysis do not seem to obey any order based on relatedness, isolates do appear to group together based on polysaccharide production phenotypes (Fig. 1). This clustering of phenotypes suggests a genetic association. Studies are ongoing to determine the genetic basis for the mucoid phenotype of these isolates, outside the known mechanisms. Coinfection has a similar pattern; isolates from coinfection cluster together (Table 1, near the top) and those not from coinfection cluster together (Table 1, near the bottom), suggesting a genetic association between S. aureus isolates obtained from the clinical microbiology laboratory at the same time as P. aeruginosa.

There was one connection between phylogeny and phenotypes that was notably absent; there was no observed relationship between the coculture interaction group and phylogeny. As seen in Table 1 and Fig. 1, there is an instance where groups cluster together; specifically, 2 out of 4 group 3 (neither kill) isolates cluster together. But group 1 (nonmucoid kills) and group 2 (both kill) isolates appear well distributed throughout the phylogeny. This suggests that what makes these isolates a specific coculture group is more complex than originally anticipated.

### Longitudinal isolates.

Some of our isolates were longitudinal; they came from the same individual with CF (“Patient ID” column of Table 1 and Fig. 1) and were collected over a period of time. In both Table 1 and Fig. 1, the longitudinal isolates cluster together in the phylogeny, suggesting that these isolates likely all originated from one initial infecting isolate or at least isolates that are very closely related. As expected, many of their phenotypes were similar. For example, most isolates collected from the same individual had the same hemolysis phenotype (Table 1 and Fig. 1, “Rabbit_hemolysis” and “Sheep_hemolysis” columns). However, one patient, CFBR105, provided four isolates in group 1 (nonmucoid kills), and two isolates in group 2 (both kill) collected over 282 days. The group 2 isolates were the last two collected, suggesting a possible transition to intolerance to mucoid P. aeruginosa under selection. However, there were no genetic changes shared between the later isolates (CFBR_32 and CFBR_33) that were absent in the earlier isolates (Table S1; Text S1). This suggests that the genetic basis of P. aeruginosa tolerance is likely complex. Isolates from patient CFBR105 also produce differing amounts of polysaccharide (Table 1), which may represent an adaption to the CF lung over time. However, an alternate hypothesis is that these isolates were always present in the lung growing in a population but that, during collection, only a single colony was chosen, possibly representing only a fraction of the diversity within the infecting population at the time of sampling.

10.1128/mBio.00735-20.1 TEXT S1 Supplemental results of longitudinal isolate mutation analysis. Download Text S1, DOCX file, 0.02 MB. Copyright © 2020 Bernardy et al. 2020 Bernardy et al. This content is distributed under the terms of the Creative Commons Attribution 4.0 International license.

10.1128/mBio.00735-20.7 TABLE S1 Mutations in ST632 isolates from patient CFBR105. Download Table S1, DOCX file, 0.02 MB. Copyright © 2020 Bernardy et al. 2020 Bernardy et al. This content is distributed under the terms of the Creative Commons Attribution 4.0 International license.

### Hemolysis and polysaccharide production were common among our isolates.

Toxins are an important part of S. aureus virulence, and alpha toxin has been suggested to be important for pulmonary infection in a CF mouse model (74). Consistent with this observation, most of the S. aureus clinical isolates tested produced hemolytic toxins, determined by their ability to completely hemolyze both rabbit and sheep blood. Interestingly, only 10 isolates (15.4%) were unable to hemolyze either blood agar. The ubiquity of hemolytic activity in these isolates suggests that S. aureus toxicity may be important in CF infection (37) or at least that it is not selected against.

S. aureus polysaccharide production has also been implicated to be important for chronic colonization in the CF lung (64). Qualitative phenotypic characterization of polysaccharide production following plating on CRA in this study shows that, consistently with this hypothesis, most isolates were capable of producing polysaccharide (both normal producers and overproducers). Twenty isolates (30.8%) were characterized as nonproducers due to their red color and smooth appearance on CRA plates. These isolates may have other mechanisms for attachment and biofilm production outside this specific polysaccharide, may live within a community that can compensate for that missing phenotype, or may benefit in some other way by not adhering to surfaces. Overall, we conclude that polysaccharide production was common among these clinical isolates, suggesting that this factor is important in CF lung infection.

### Group 1 isolates may come from initial infection.

While it is generally understood that P. aeruginosa kills S. aureus *in vitro* (14–18, 68, 75) and is hypothesized to also do so *in vivo*, these previous studies were performed on a small number of strains and focused on how P. aeruginosa kills S. aureus. Our studies here allow us to determine if P. aeruginosa killing S. aureus is typical of CF isolates. Our group previously showed that JE2, an isolate from a wound infection, was killed by nonmucoid PAO1 but survived when cocultured with mucoid PAO1 and subsequently discussed the mechanism behind this conclusion (18); therefore, we examined if S. aureus CF isolates behaved similarly. The majority of our isolates (46 of 65), including JE2, were killed by nonmucoid PAO1 only, and we subsequently called these our group 1 (nonmucoid kills) isolates. While it is not surprising that these isolates behaved this way due to previously defined mechanisms discussed in the work of Limoli et al. (18), it is interesting that isolates from lung infection behave the same as an isolate from a wound infection with regard to interaction with P. aeruginosa. Remarkably, when we looked at group 1 (nonmucoid kills) isolates in relation to other data collected in this study (Table 1), many were from younger patients and were sensitive to aminoglycosides and glycopeptides. This observation suggests that these isolates may be from the initial stages of infection. There is a known switch in predominance of infection from S. aureus in childhood to P. aeruginosa in adults with CF in the United States which aligns with our observation, because S. aureus isolates from initial infection could be sensitive to P. aeruginosa which would allow for this transition to occur. These trends remain when you instead look at the fold change values in Table 1 instead of the coculture group. Therefore, while assigning groups may seem subjective due to a chosen fold change cutoff, the conclusions still stand when observing fold change values alone.

### Group 2 isolates were incapable of hemolysis but more resistant to antibiotics.

While most isolates were in group 1 (nonmucoid kills), we recognized a set of isolates that were killed by both nonmucoid and mucoid PAO1, denoted group 2 (15 out of 65 isolates; both kill). This observed phenotype was surprising to us based on how often these two bacteria are thought to coinfect the CF lung. It is likely that these isolates have not come in direct contact with P. aeruginosa and therefore have not had a need to develop defensive strategies. These isolates may also have other mutations that increase their fitness in CF lung that coincidentally led to them being less competitive with P. aeruginosa. In line with this hypothesis, half of the isolates that were incapable of hemolyzing either type of blood agar (Rabbit −/Sheep −) (Table 2) were in coculture group 2 (both kill). Therefore, it is tempting to speculate that these isolates may have lost some virulence phenotypes, resulting in lack of hemolysis and increased susceptibility to killing by P. aeruginosa. While looking at group 2 (both kill) isolates in Table 1, we noticed that many were from older patients and were resistant to methicillin (MRSA), aminoglycosides, and glycopeptides. As stated above, these trends were still present when we looked at fold change values instead of the coculture group. These observations may suggest that group 2 isolates (both kill) come from chronic infection due to increases in the antibiotic resistance and the age of the patient from which they were collected. There is also a study suggesting an inverse relationship between toxin production and the ability to cause infections, with low-cytotoxicity isolates causing more fatal infections (76). These data support our hypothesis that these isolates might cause chronic infection because many group 2 isolates (both kill) were negative for hemolysis. As previously mentioned, some of our longitudinal isolates switched to group 2 over time in the same patient (Table S1), consistent with these data. Therefore, we might be observing a S. aureus adaptation over time in the CF lung where the isolate loses expression of virulence factors, similarly to P. aeruginosa. It is interesting to consider that during CF lung infection, it might be more advantageous for S. aureus to retain antibiotic resistance phenotypes rather than to relinquish them to coexist with P. aeruginosa, leading to a group 2 interaction phenotype.

### Group 3 isolates were coinfected with P. aeruginosa at the time of collection.

The most surprising group of isolates are those that were resistant to killing by both nonmucoid and mucoid PAO1, denoted group 3 (4 of 65 isolates; neither kills). P. aeruginosa is a potent competitor and utilizes an arsenal of extracellular products and other mechanisms to kill neighboring bacteria; therefore, it is interesting that these S. aureus isolates have developed defensive strategies that allow them to survive coculture *in vitro*. Unsurprisingly, when investigating the metadata associated with these isolates, we discovered that all four of these isolates, all of which came from different individuals, were coinfected with P. aeruginosa at the time of collection. Of the 46 group 1 isolates (nonmucoid kills), 20 (43.4%) were coinfected with P. aeruginosa at the time of collection, while 7 of the 15 (46.7%) group 2 (both kill) isolates were from coinfected individuals, signifying that coinfection was most important for group 3 (neither kills) isolates. While much work has been done to show that P. aeruginosa and S. aureus do not appear to come in direct contact during an established infection in a chronic-wound model (77, 78), whether this holds true in the context of CF has not been clearly shown. In CF, it is possible that during initial infection, there is interaction between these two microbes but that they eventually separate and create a spatial structure due to their antagonistic interaction, which follows a well-studied ecological theory (79). It is also possible that these S. aureus isolates obtained other fitness benefits from genetic changes that allow for coexistence with P. aeruginosa.

### Diversity of S. aureus CF isolates.

Based on the analysis performed here, we conclude that the U.S. S. aureus CF clinical isolates surveyed here are not from clonal lineages that transmit between CF patients but instead are from multiple independent colonization events. Not only do they vary in virulence phenotypes, but also in their interactions with P. aeruginosa. These variations may be due to inherent differences during initial infection or evolutionary changes in response to their environment, both that of the CF lung but also of the presence of other pathogens like P. aeruginosa or other members of the lung microbiome (80). Most isolates retained virulence-associated phenotypes, namely, hemolytic activity and the mucoid phenotype, after infecting the CF lung. The mucoid phenotype may aid in adhesion and can protect S. aureus from immune cell attack, so it was not surprising to find that many of our isolates were mucoid.

Many studies have previously shown that P. aeruginosa kills S. aureus in a variety of *in vitro* experiments. Some have also shown P. aeruginosa isolates that cannot kill S. aureus; however, a widespread examination of S. aureus isolates and their ability to withstand P. aeruginosa attack had not been performed prior to this study. While we have not yet identified the S. aureus mechanisms involved in the diversity of interaction with P. aeruginosa, we have ruled out some specific genotypes and phenotypes. We conclude that this interaction is complex and multifactorial. There were no striking phenotypes or genotypes that were specific or unique for each coculture interaction group. We are currently further investigating these S. aureus isolates for genetic factors or phenotypes responsible for the varying interaction with P. aeruginosa.

Interestingly, none of the S. aureus isolates discussed in this study were small-colony variants, which has been shown to be a known adaptation to the CF lung environment (27–30). Some became small-colony variants after being challenged with P. aeruginosa, but this was not consistent, and the strains quickly reverted when restreaked alone on isolation agar for S. aureus. This leads us to a limitation of our study; we collected only single isolates from a patient, which may have led us to lose small-colony variants as well as to not fully understand the prevalence of the phenotypes described in this study. We appreciate that bacteria live in populations in the CF lung and that many of these phenotypes may live together. Phenotypes that involve extracellular products, such as polysaccharide and toxin production, may allow for *trans*-complementation in a community where isolates incapable of performing those actions may still benefit from other isolates capable of producing these products. Therefore, in the future, we hope to obtain multiple colonies from a single patient to better understand the diversity of S. aureus inside a single individual and expand the P. aeruginosa strains used in our coculture test.

S. aureus is the most prevalent cause of lung infection in individuals with CF in the United States, yet a small number of large-scale studies combining genomic and phenotypic data had been performed before this study. Understanding the diversity of these isolates and how specific phenotypes and genotypes connect to patient health is paramount to the development of more effective treatments for CF respiratory infections. If we can provide clinical microbiology labs with a list of specific S. aureus traits to monitor in order to prevent coinfection between P. aeruginosa and S. aureus and the associated health decline, we could make a huge impact on the health of individuals with CF. Outside of lung infections, MRSA causes a substantial number of infections at all body sites and is recognized as a significant threat by the CDC. We have identified a subset of isolates that are sensitive to attack by other bacteria. If we can identify what kills these bacteria or what genes make them sensitive, it could provide new treatment options for these notoriously hard-to-treat infections. Overall, our work contributes to a better understanding of the diversity of S. aureus and how it adapts in CF lung infections.

## MATERIALS AND METHODS

### Bacterial strains and growth conditions.

The S. aureus isolates used in the study are listed in Table 1. These isolates are renamed in this publication in order to simplify their names. Therefore, we have provided Table S2 in the supplemental material outlining this name change from this publication with their sequences in Bernardy et al. (38). P. aeruginosa strains used were laboratory strain nonmucoid PAO1 (81) and mucoid PAO1 containing the *mucA22* allele, also known as PDO300 (82). P. aeruginosa and S. aureus were grown in lysogeny broth (LB) and Trypticase soy broth (TSB), with 1.5% agar for solid medium. Selective media for P. aeruginosa was *Pseudomonas* isolation agar (PIA; BD Difco), while selective medium for S. aureus was Trypticase soy agar (TSA; BD BBL) with 7.5% NaCl, called *Staphylococcus* isolation agar (SIA).

10.1128/mBio.00735-20.8 TABLE S2 Isolate name change from Bernardy et al. (38). Download Table S2, DOCX file, 0.02 MB. Copyright © 2020 Bernardy et al. 2020 Bernardy et al. This content is distributed under the terms of the Creative Commons Attribution 4.0 International license.

### Whole-genome phylogeny, virulence factor gene search, and longitudinal isolates analysis.

The genomes were processed with the Staphopia (42) analysis pipeline. Each sample was assembled with SPAdes (83) and annotated with Prokka (84). ARIBA (58) was used to match sequence data from each project against the MEGARES antibiotic resistance database (85) to determine resistance or sensitivity to antibiotic classes. Roary (86) was used to determine the pan-genome and to create a core genome alignment with MAFFT (87). Recombination was identified in the core genome alignment with ClonalFrameML (88) and masked with maskrc-svg (https://github.com/kwongj/maskrc-svg). A phylogenetic tree based on the masked core genome alignment was created with IQ-TREE (89–91) with automatic substitution model selection and 1,000 ultrafast bootstrap replicates. The log likelihood score for the consensus tree constructed from 1,000 bootstrap trees was −2,575,231. We used FastANI to obtain pairwise average nucleotide identity estimates (92). Illumina data of isolates from patient CFBR105 were compared against the complete genome of the first isolate, CFBR_29 (RefSeq accession number NZ_CP031779.1 [38], PRJNA480016, AAB80783.1, AAB63265.1, AAB63268.1, and AAG03056.1), using breseq (93).

The core VFDB proteome (http://www.mgc.ac.cn/VFs/Down/VFDB_setA_pro.fas.gz) was downloaded on 13 April 2020 and Staphylococcus aureus selected. The proteins were used to query the database of assembled contigs of the genomes of this study using tblastn. The percentage identity of the top match for each protein with a match of ≥100 amino acids and ≥40% was determined. If the match fell below these thresholds, the protein was determined to be absent in the isolate.

### Genotypic characterization of virulence phenotypes.

Alpha and beta toxin sequences (*hla* and *hlb*, respectively) were extracted from the hemolysis-positive S. aureus JE2 reference genome (accession number GCA_002085525.1) and were queried against the S. aureus CF clinical isolate genomes using BLAST 2.9.0 (94). A similar strategy was adopted for *agr* typing, for which AgrD sequences for the 4 *agr* groups were used as references (GenBank accession numbers AAB80783.1, AAB63265.1, AAB63268.1, and AAG03056.1). Hits with >95% amino acid sequence identity and >90% query coverage were considered to be positive for the presence of alpha/beta toxin or the respective *agr* type.

Staphylococcal protein A (*spa*) type repeat successions and sequences corresponding to individual repeats were downloaded from the Ridom Spa Server (52). These files were then combined to create a FASTA file having the complete sequence for 18,915 *spa* types. These sequences were then converted to a BLAST database and used to query our S. aureus genomes. Because *spa* types are assigned based on repeat sequence identity and the number of repeats, we assigned the longest *spa* sequence having a 100% sequence match and 100% query coverage as the *spa* type for a given sample.

### Hemolysis assays.

S. aureus CF clinical isolates and the lab isolate JE2 were plated on rabbit blood and sheep blood agar plates. Briefly, wooden sticks were placed in a cryovial with a frozen stock of the chosen isolate and then gently touched to the surface of the chosen blood agar plate. Plates were incubated at 37°C for 24 h, and the presence or absence of clear hemolysis was recorded. After this, the plates were incubated again at 4°C for 24 h, and clear hemolysis presence/absence was recorded again. For ease, each isolate was scored as “+” if clear hemolysis was detected or “–” for no clearing (not hemolyzing blood). All isolates tested grew on both types of plates.

### Phenotypic characterization of polysaccharide production.

Each S. aureus CF clinical isolate, along with positive (the MN8 wild type and an MN8 mucoid strain which had a 5-bp deletion in the *ica* operon) and negative (MN8 Δ*ica*, which has the entire *ica* operon deleted) controls for polysaccharide production (provided by Gerald B. Pier, Brigham & Women’s Hospital, Harvard Medical School) (66), was streaked for single colonies on Congo red agar (CRA) plates. CRA was made as previously described (67). We combined 18.5 g Oxoid brain heart infusion broth, 25 g sucrose, and 5 g agar in 500 ml of distilled water and autoclaved it. After it cooled to ∼55°C, we then added 8 ml of Congo red dye stock solution (made by dissolving 5 g into 100 ml and autoclaving). Briefly, wooden sticks were placed in cryovials with a frozen stock of each isolate and then streaked across CRA. Plates were incubated at 37°C for 24 h. Results were interpreted as previously described (64, 95–97): black and deep-red smooth colonies were considered to be normal polysaccharide-producing strains (like wild-type MN8), red smooth colonies were considered to be nonproducers (like MN8 Δ*ica*), and rough colonies of any color (like mucoid MN8) were considered to be overproducers.

### Coculture assay.

To monitor the interaction between P. aeruginosa and the S. aureus isolates in this study, we performed a quantitative coculture assay using either nonmucoid or mucoid P. aeruginosa strain PAO1. Briefly, wooden sticks were placed in cryovials with frozen stocks of nonmucoid and mucoid PAO1 and then streaked for single colonies onto PIA, while S. aureus isolates were streaked onto SIA. Both were incubated at 37°C overnight. Single colonies were selected and then grown in liquid LB at 37°C overnight. These cultures of P. aeruginosa and S. aureus were then back-diluted to an optical density of 0.05 and mixed in a 1:1 ratio with each other or with sterile broth as “alone” controls. Ten microliters of each mixture was placed onto a 0.45-μm filter on a TSA plate and incubated at 37°C for 24 h. After incubation, filters were removed using sterile forceps, and bacteria were resuspended in 1.5 ml of sterile LB before serial dilution and plating onto PIA and SIA. After incubation at 37°C overnight, colonies were counted and numbers of CFU per milliliter calculated. Fold change of S. aureus was calculated by dividing the CFU per milliliter of S. aureus grown with nonmucoid PAO1 or with mucoid PAO1 (average CFU per milliliter with standard deviation error bars in Fig. S5) over the CFU per millilter of each S. aureus isolate grown alone (average CFU per milliliter with standard deviation error bars in Fig. S4). A fold change of <10-2 was considered to indicate significantly killing.

10.1128/mBio.00735-20.6 FIG S5 The average CFU per milliliter used to calculate fold change show little deviation among technical triplicate values. The average CFU per milliliter of each S. aureus isolate grown with nonmucoid or mucoid PAO1 during coculture assay (these exact numbers were used to calculate the fold changes in Fig. 2). Error bars show standard deviations from technical triplicates. Download FIG S5, EPS file, 1.5 MB. Copyright © 2020 Bernardy et al. 2020 Bernardy et al. This content is distributed under the terms of the Creative Commons Attribution 4.0 International license.

### Availability of data.

Raw Illumina reads available under BioProject accession number PRJNA480016 were used in this study.
