# Supplementary material for: Genotypic and Phenotypic Diversity of Staphylococcus aureus Isolates from Cystic Fibrosis Patient Lung Infections and Their Interactions with Pseudomonas aeruginosa
Source: mBio. 2020 Jun 23;11(3):e00735-20. doi: 10.1128/mBio.00735-20 (PMC7315118; doi:10.1128/mBio.00735-20)
Supplement: TEXT S1 [file mBio.00735-20-s0001.docx]

**Supplemental Results: Longitudinal isolate mutation analysis**

We collected six *S. aureus* isolates from patient CFBR-105 over a period of 282 days. We were able to close the genome of the first isolate, CFBR_29, after additional Nanopore sequencing. Illumina reads from isolates sampled at later dates were then mapped against the complete genome of the first isolate, CFBR_29, using breseq [1]. This genome was described in [2] (under the previous name “CFBR_EB_Sa105”) and was submitted to NCBI as accession NZ_CP031779.1. Supplemental Table S1 shows all of the single nucleotide polymorphisms (SNPs) and insertions or deletions (INDELs) present in each isolate when compared to CFBR_29. The position in CFBR_29 full genome is shown as well as the specific mutation (Table S1). Of the 25 mutations present between CFBR_29 and the later isolates (CFBR_16, CFBR_30, CFBR_31, CFBR_32, and CFBR_33), 22 were SNPs and 3 were INDELs. For the later isolates, the bottom row gives the number of days after CFBR_29 was isolated that each of the other five isolates were sampled from the same patient (Table S1). The presence or absence of each mutation for each isolate is represented by a “+” or “-”, respectively. There were some isolates that did not have proper coverage over certain genes making it difficult to determine if the mutation of interest was present or absent, resulting in a “?” on Table S1. The effect of the mutation is outlined in Table S1 as synonymous SNPs (in green text), nonsynonymous SNPs (in blue text), or details of location of INDELs or intergenic SNPs/INDELs (in black text).

CFBR_32 and CFBR_33 were found to be in coculture Group 2 (both kill), while the other isolates were in Group 1 (nonmucoid kills), and this is indicated by the thick border between these isolates. As shown in Table S1, there were no mutations only present in either coculture group while absent in the other. This suggests that phenotype that results in tolerance to killing by *P. aeruginosa* is complex and likely due to more than a few point mutations. However, CFBR_32 has non-synonymous changes in two genes, Sapep Mn2+-metalloprotease and a D‑amino‑acid transaminase, not shared with other isolates. CFBR_33 unique changes include four intergenic SNPs, an early termination in adhesin SdrE [3] and non-synonymous changes in three other genes. Further experimental work is needed determine which of these mutations (or combination of mutations) causes the phenotype.

References

1. Deatherage DE, Barrick JE. Identification of mutations in laboratory-evolved microbes from next-generation sequencing data using breseq. Methods Mol Biol. 2014;1151:165-88.

2. Bernardy EE, Petit RA, 3rd, Moller AG, Blumenthal JA, McAdam AJ, Priebe GP, et al. Whole-Genome Sequences of Staphylococcus aureus Isolates from Cystic Fibrosis Lung Infections. Microbiol Resour Announc. 2019;8(3).

3. Josefsson E, McCrea KW, Ni Eidhin D, O'Connell D, Cox J, Hook M, et al. Three new members of the serine-aspartate repeat protein multigene family of Staphylococcus aureus. Microbiology. 1998;144 ( Pt 12):3387-95.
