# Supplementary material for: Genotypic and Phenotypic Diversity of Staphylococcus aureus Isolates from Cystic Fibrosis Patient Lung Infections and Their Interactions with Pseudomonas aeruginosa
Source: mBio. 2020 Jun 23;11(3):e00735-20. doi: 10.1128/mBio.00735-20 (PMC7315118; doi:10.1128/mBio.00735-20)
Supplement: TABLE S1 [file mBio.00735-20-st001.docx]

**Table S1. Mutations in ST632 isolates from patient CFBR-105.**

| **CFBR_29 genome position** | **Mutation** | **CFBR_16** | **CFBR_30** | **CFBR_31** | **CFBR_32** | **CFBR_33** | **Effect** | **CFBR_29 gene** | **Gene description** |
| --- | --- | --- | --- | --- | --- | --- | --- | --- | --- |
| 79,655 | AAAAAAAA→AAAAAAA | + | + | - | - | - | coding (at 346 out of 558 nt) | *D1F65_RS00375* | K(+)‑transporting ATPase subunit C |
| 194,331 | C→T | + | + | - | - | - | G88G (GGC→GGT) | *D1F65_RS00920* | MFS transporter |
| 248,616 | C→T | + | + | - | - | - | A344V (GCG→GTG) | *D1F65_RS01120* | Gfo/Idh/MocA family oxidoreductase |
| 481,778 | C→T | + | + | - | - | - | intergenic (at +314 or ‑18) | between *D1F65_RS02330* and *aaa* | between dipeptide ABC transporter glycylmethionine‑binding lipoprotein and autolysin/adhesin Aaa |
| 519,637 | T→C | - | - | + | + | - | H71H (CAT→CAC) | *rsmA* | 16S rRNA (adenine(1518)‑N(6)/adenine(1519)‑N(6))‑ dimethyltransferase RsmA |
| 577,523 | T→C | - | - | + | + | - | L71S (TTA→TCA) | *nusG* | transcription termination/antitermination protein NusG |
| 615,694 | G→A | - | - | - | - | + | A296T (GCA→ACA) | *sdrE* | MSCRAMM family adhesin SdrE |
| 717,628 | A→G | + | + | - | - | - | M309V (ATG→GTG) | *D1F65_RS03515* | inorganic phosphate transporter |
| 803,567 | C→T | + | + | + | + | + | S209S (AGC→AGT) | *D1F65_RS03965* | undecaprenyl/decaprenyl‑phosphate alpha‑N‑acetylglucosaminyl 1‑phosphate transferase |
| 966,130 | C→G | + | + | + | + | + | R3T (AGA→ACA) | *trpS* | tryptophan‑‑tRNA ligase |
| 1,003,279 | G→T | - | - | - | - | + | intergenic (at +169 or ‑63) | between *D1F65_RS04980* and *D1F65_RS04985* | between CPBP family intramembrane metalloprotease and putative holin‑like toxin |
| 1,089,169 | C→T | ? | ? | + | + | ? | G29E (GGA→GAA) | *D1F65_RS05435* | ISL3‑like element IS1181 family transposase |
| 1,400,215 | C→T | - | - | + | - | - | S683L (TCA→TTA) | *parC* | DNA topoisomerase IV subunit A |
| 1,565,214 | T→A | + | + | - | - | - | pseudogene (at 17 out of 99 nt) | *D1F65_RS07795* | GNAT family N‑acetyltransferase |
| 1,611,258 | G→A | + | + | - | - | - | T112I (ACA→ATA) | *accB* | acetyl‑CoA carboxylase biotin carboxyl carrier protein |
| 1,827,456 | Δ1 bp | - | - | - | - | + | intergenic (‑69/+285) | between *D1F65_RS09160* and *aroF* | between hypothetical protein and 3‑deoxy‑7‑phosphoheptulonate synthase |
| 1,844,342 | C→A | - | - | - | + | - | G28V (GGC→GTC) | *D1F65_RS09235* | D‑amino‑acid transaminase |
| 1,845,573 | G→C | - | - | - | + | - | P89A (CCT→GCT) | *sapep* | Mn(2+)‑dependent dipeptidase Sapep |
| 1,910,394 | GAAAGAAA→GAAA | + | + | - | - | - | coding (at 403‑406 out of 792 nt) | *D1F65_RS09575* | DUF1828 domain‑containing protein |
| 2,070,021 | T→C | + | + | - | - | - | I288V (ATC→GTC) | *D1F65_RS10570* | YeeE/YedE family protein |
| 2,277,351 | A→G | - | - | - | - | + | Y34H (TAT→CAT) | *D1F65_RS11665* | type Z 30S ribosomal protein S14 |
| 2,340,638 | T→C | - | - | - | - | + | intergenic (at ‑27 or +576) | between *D1F65_RS12060* and *D1F65_RS12065* | between formate dehydrogenase subunit alpha and transcriptional regulator |
| 2,522,711 | T→C | - | - | - | - | + | D175G (GAC→GGC) | *D1F65_RS13040* | tandem‑type lipoprotein |
| 2,558,608 | G→A | - | - | + | + | - | F329F (TTC→TTT) | *D1F65_RS13195* | MFS transporter |
| 2,636,921 | G→T | - | - | - | - | + | intergenic (at ‑151 or +190) | between *D1F65_RS13605* and *D1F65_RS13610* | TIGR04197 family type VII secretion effector/fructosamine kinase family protein |
| 2,770,781 | G→A | - | - | - | - | + | G61S (GGT→AGT) | *D1F65_RS14230* | peptide resistance ABC transporter ATP‑binding subunit VraD |
| Days after CFBR_29 | | 84 | 84 | 174 | 246 | 282 |  |  |  |
