# Supplementary material for: Genotypic and Phenotypic Diversity of Staphylococcus aureus Isolates from Cystic Fibrosis Patient Lung Infections and Their Interactions with Pseudomonas aeruginosa
Source: mBio. 2020 Jun 23;11(3):e00735-20. doi: 10.1128/mBio.00735-20 (PMC7315118; doi:10.1128/mBio.00735-20)
Supplement: TABLE S2 [file mBio.00735-20-st002.docx]

| **Table S2. Isolate name change from Bernardy et al. [38].** | |  |
| --- | --- | --- |
| **Isolate name in this publication** | **Isolate name in Bernardy et al. [38]** | **NCBI Accession number** |
| JE2 | JE2 | SAMN09847868 |
| CFBR_01 | CFBRSa03 | SAMN09847832 |
| CFBR_02 | CFBRSa04 | SAMN09847836 |
| CFBR_03 | CFBRSa05 | SAMN09847841 |
| CFBR_04 | CFBRSa06 | SAMN09847844 |
| CFBR_05 | CFBRSa07 | SAMN09847850 |
| CFBR_06 | CFBRSa21 | SAMN09847840 |
| CFBR_07 | CFBRSa22 | SAMN09847842 |
| CFBR_08 | CFBRSa23 | SAMN09847846 |
| CFBR_09 | CFBRSa24 | SAMN09847849 |
| CFBR_10 | CFBRSa25 | SAMN09847834 |
| CFBR_11 | CFBRSa26 | SAMN09847823 |
| CFBR_12 | CFBRSa27 | SAMN09847845 |
| CFBR_13 | CFBRSa28 | SAMN09847848 |
| CFBR_14 | CFBRSa29 | SAMN09847856 |
| CFBR_15 | CFBRSa30 | SAMN09847835 |
| CFBR_16 | CFBRSa47 | SAMN09847830 |
| CFBR_17 | CFBRSa48 | SAMN09847824 |
| CFBR_18 | CFBRSa49 | SAMN09847831 |
| CFBR_19 | CFBRSa50 | SAMN09847833 |
| CFBR_20 | CFBRSa51 | SAMN09847837 |
| CFBR_21 | CFBRSa66A | SAMN09847838 |
| CFBR_22 | CFBRSa66B | SAMN09847839 |
| CFBR_23 | CFBRSa70 | SAMN09847843 |
| CFBR_24 | CFBRSa74 | SAMN09847847 |
| CFBR_25 | CFBR_EB_Sa101 | SAMN09847819 |
| CFBR_26 | CFBR_EB_Sa102 | SAMN09847820 |
| CFBR_27 | CFBR_EB_Sa103 | SAMN09847821 |
| CFBR_28 | CFBR_EB_Sa104 | SAMN09847822 |
| CFBR_29 | CFBR_EB_Sa105 | SAMN09847825 |
| CFBR_30 | CFBR_EB_Sa108 | SAMN09847826 |
| CFBR_31 | CFBR_EB_Sa110 | SAMN09847827 |
| CFBR_32 | CFBR_EB_Sa112 | SAMN09847828 |
| CFBR_33 | CFBR_EB_Sa114 | SAMN09847829 |
| CFBR_34 | CFBR_EB_Sa116 | SAMN09847851 |
| CFBR_35 | CFBR_EB_Sa117 | SAMN09847852 |
| CFBR_36 | CFBR_EB_Sa118 | SAMN09847853 |
| CFBR_37 | CFBR_EB_Sa119 | SAMN09847854 |
| CFBR_38 | CFBR_EB_Sa121 | SAMN09847855 |
| CFBR_39 | CFBR_EB_Sa122 | SAMN09847857 |
| CFBR_40 | CFBR_EB_Sa123 | SAMN09847858 |
| CFBR_41 | CFBR_EB_Sa125 | SAMN09847859 |
| CFBR_42 | CFBR_EB_Sa126 | SAMN09847860 |
| CFBR_43 | CFBR_EB_Sa127 | SAMN09847861 |
| CFBR_44 | CFBR_EB_Sa129 | SAMN09847862 |
| CFBR_45 | CFBR_EB_Sa130 | SAMN09847863 |
| CFBR_46 | CFBR_EB_Sa131 | SAMN09847864 |
| CFBR_47 | CFBR_EB_Sa133 | SAMN09847865 |
| CFBR_48 | CFBR_EB_Sa135 | SAMN09847866 |
| CFBR_49 | CFBR_EB_Sa138 | SAMN09847867 |
| BCH-SA-01 | BCH-SA-01 | [SAMN09847804](https://www.ncbi.nlm.nih.gov/biosample/SAMN09847804) |
| BCH-SA-02 | BCH-SA-02 | [SAMN09847805](https://www.ncbi.nlm.nih.gov/biosample/SAMN09847805) |
| BCH-SA-03 | BCH-SA-03 | [SAMN09847806](https://www.ncbi.nlm.nih.gov/biosample/SAMN09847806) |
| BCH-SA-04 | BCH-SA-04 | [SAMN09847807](https://www.ncbi.nlm.nih.gov/biosample/SAMN09847807) |
| BCH-SA-05 | BCH-SA-05 | [SAMN09847808](https://www.ncbi.nlm.nih.gov/biosample/SAMN09847808) |
| BCH-SA-06 | BCH-SA-06 | [SAMN09847809](https://www.ncbi.nlm.nih.gov/biosample/SAMN09847809) |
| BCH-SA-07 | BCH-SA-07 | [SAMN09847810](https://www.ncbi.nlm.nih.gov/biosample/SAMN09847810) |
| BCH-SA-08 | BCH-SA-08 | [SAMN09847811](https://www.ncbi.nlm.nih.gov/biosample/SAMN09847811) |
| BCH-SA-09 | BCH-SA-09 | [SAMN09847812](https://www.ncbi.nlm.nih.gov/biosample/SAMN09847812) |
| BCH-SA-10 | BCH-SA-10 | [SAMN09847813](https://www.ncbi.nlm.nih.gov/biosample/SAMN09847813) |
| BCH-SA-11 | BCH-SA-11 | [SAMN09847814](https://www.ncbi.nlm.nih.gov/biosample/SAMN09847814) |
| BCH-SA-12 | BCH-SA-12 | [SAMN09847815](https://www.ncbi.nlm.nih.gov/biosample/SAMN09847815) |
| BCH-SA-13 | BCH-SA-13 | [SAMN09847816](https://www.ncbi.nlm.nih.gov/biosample/SAMN09847816) |
| BCH-SA-14 | BCH-SA-14 | [SAMN09847817](https://www.ncbi.nlm.nih.gov/biosample/SAMN09847817) |
| BCH-SA-15 | BCH-SA-15 | [SAMN09847818](https://www.ncbi.nlm.nih.gov/biosample/SAMN09847818) |
